# Analytical models representing X-ray form factors of ions

**DOI:** 10.1107/S2053273323010550

**Published:** 2024-01-01

**Authors:** Gunnar Thorkildsen

**Affiliations:** a Department of Mathematics and Physics, University of Stavanger, N-4036 Stavanger, Norway; Institute of Crystallography - CNR, Bari, Italy

**Keywords:** X-ray form factors, inverse Mott–Bethe formula, analytical representations, ions

## Abstract

Parameters in analytical models for X-ray form factors of ions *f*_0_(*s*), based on the inverse Mott–Bethe formula involving a variable number of Gaussians, are determined for a wide range of published data sets {*s*, *f*_0_(*s*)}. The models reproduce the calculated form-factor values close to what is expected from a uniform statistical distribution with limits determined by their precision. For different ions associated with the same atom, the number of Gaussians in the models decreases with increasing net positive charge.

## Abstract 2

Analytical representations of X-ray form factors for ions are examined based on the inverse Mott–Bethe formula. Twelve sources of form-factor data spanning the period 1961–2023 and representing various ranges and grids in sinθ/λ and different precisions are analysed. Generally, 96.1% of all form factors are exactly reproduced by the analytical models.

## 1. Introduction

In a previous paper (Thorkildsen, 2023 ▸), hereafter denoted GT-I, the inverse Mott–Bethe formula was successfully applied to model X-ray form-factor data for neutral atoms. Here, the application of a modified algorithm to model form-factor data for ions is reported. As in GT-I, data from a number of sources have been examined to verify the versatility of the analysis: Watson & Freeman (1961 ▸), Ibers (1962 ▸), Cromer *et al.* (1963 ▸), Cromer & Mann (1968 ▸), Doyle & Turner (1968 ▸), Cromer & Waber (1974 ▸) and Maslen *et al.* (1992 ▸), Rez *et al.* (1994 ▸), Wang *et al.* (1996 ▸), Macchi & Coppens (2001 ▸), Yonekura *et al.* (2018 ▸), Olukayode *et al.* (2023*b* ▸), and Volkov (2023 ▸).

## 2. Formulas

The basic expression used to model form-factor data for ions is 

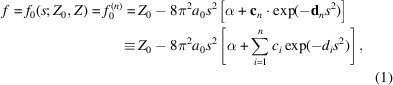

with 

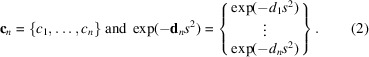

This model is denoted MB[*n*G + α]. *a*_0_ is the Bohr radius and 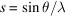 . *n*, giving the number of Gaussians in the model, is treated as a variable. *Z*_0_ is interpreted as the number of electrons and *Z* is the atomic number of the charged atom in question. Δ*Z* = *Z* − *Z*_0_ is thus the net ionic charge. The traditional model for form factors, referred to as S[*n*G + *c*], is 

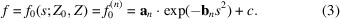

Here *n* has been treated as a constant. *n* = 2, …, 6 have been reported in the literature.

## 3. Method

As in GT-I, the fitting procedure here is performed using the *Mathematica* function NonlinearModelFit (Wolfram Research, 2023 ▸). All observations are associated with unit weights. The analysis leading to the final values of the parameters of the model, {α, *c*_1_, …, *c*_*n*_, *d*_1_, …, *d*_*n*_}, is a slightly changed version of the one reported in GT-I. This has affected primarily the *Search* and *Expand* modules. *Repair* has become obsolete.

(i) *Search*: The *Search* module represents the initial part of the procedure and is usually performed only once involving a small number of Gaussians. The random-number generator RandomReal returns initial values for the *d* parameters (in units of Å^2^), here shown for the default case of three Gaussians: 

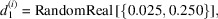




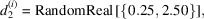




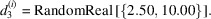

The value 1.0 Å is associated with α^(*i*)^ and 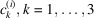 . Refinements are then conducted to obtain parameter sets for model MB[3G + α] for all ions in the data set.

(ii) *Expand*: Form-factor data sets for ions normally exhibit a greater span in the number of Gaussians, which appears in the final analytical models, than was found in the work on neutral atoms. Thus the *Expand* part of the analysis, *i.e.* MB[*n*G + α] → MB[(*n* + 1)G + α], which aims to increase in steps the number of parameters in the model by two, giving a better fit to the original data, has been slightly altered: 

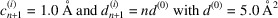

are appended to the parameters obtained using *n* Gaussians in the previous step of the refinements (the first step is the *Search* process). Together they represent the new sets of initial values. Subsequently, refinements are conducted for all (remaining) ions in the set. If the refinement for some ions fails, *Expand* is repeated, first with *d* ^(0)^ = 2.5 Å^2^ and then, if necessary, with *d* ^(0)^ = 10.0 Å^2^. The ratios among the *d* ^(0)^ values, 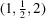 , are usually kept fixed, but the actual values have been the subject of some trial and error. If no new model MB[(*n* + 1)G + α] is obtained for a given ion, the model MB[*n*G + α] is regarded as the final representation.

(iii) General comments: In cases where the ratio 

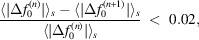

model MB[*n*G + α] is used as the final one. An improvement of less than 2% in the mean absolute error does not warrant an additional Gaussian in the model (this also manifests itself in increasing parameter uncertainties). The constraint for the final set of *d* values is updated: 

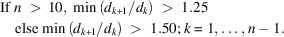

Otherwise, conditions to be satisfied by the parameters are as in GT-I. Relative parameter uncertainties are always assessed as part of the final verification of the models. In a very few cases this may result in choosing models from a previous step, having one less Gaussian, as the definitive ones.

## 4. Analyses

The X-ray form-factor data sets covered in this work are denoted as follows: WFi (Watson & Freeman, 1961 ▸), ITiiii (Ibers, 1962 ▸), CLWi (Cromer *et al.*, 1963 ▸), CMi (Cromer & Mann, 1968 ▸), DTi (Doyle & Turner, 1968 ▸), ITCi (Cromer & Waber, 1974 ▸; Maslen *et al.*, 1992 ▸), RRGi (Rez *et al.*, 1994 ▸), WSBJi (Wang *et al.*, 1996 ▸), MCi (Macchi & Coppens, 2001 ▸), Yetali (Yonekura *et al.*, 2018 ▸), OFFV1i (Olukayode *et al.*, 2023*b* ▸) and OFFV2i (Volkov, 2023 ▸). Note that form-factor data from Watson & Freeman (1961 ▸) are partly included in Ibers (1962 ▸).

OFFVi is used for properties common to OFFV1i and OFFV2i. The data set for neutral atoms provided by Volkov and described in GT-I is here denoted by OFFV2.

Complete lists of the species incorporated in the data sets are given in the supporting information. The actual number of species is summarized in Table 1 ▸.

The analytical setup for each data set is comprised of model functions MB[*n*G + α] of equation (1). The number of Gaussians involved in the final models is listed in Table 2 ▸. *n* spans the interval *n* ∈ [2, 18]. Precisions, number of sampling grid points and number of form factors in the various sets are included in Table 3 ▸. A precision of 1 × 10^−5^ is assessed as a convenient practical limit and 1 × 10^−7^ as the lower limit for retaining numerical accuracy throughout the analysis (this only affects MCi and OFFV2i). Sampling grids are summarized below.

(i) WFi: The data are characterized by *s* ∈ [0.00, 1.50] Å^−1^ in a grid Δ*s* 0.00 (0.05) 0.50 Å^−1^ and 0.50 (0.10) 1.50 Å^−1^.

(ii) ITiiii: *s* ∈ [0.00, 1.90] Å^−1^ in a grid Δ*s* 0.00 (0.05) 0.40 Å^−1^ and 0.40 (0.10) 1.90 Å^−1^. However, depending on the actual sources used in the compilation by Ibers, form factors are presented in various grids, all being subsets of the one given above.

(iii) CLWi: *s* ∈ [0.00, 1.99] Å^−1^ in a grid Δ*s* 0.00 (0.01) 1.99 Å^−1^.

(iv) CMi: *s* ∈ [0.00, 1.50] Å^−1^ in a grid Δ*s* 0.00 (0.01) 1.50 Å^−1^.

(v) DTi: *s* ∈ [0.00, 6.00] Å^−1^ in a grid Δ*s* 0.00 (0.05) 0.50 Å^−1^, 0.50 (0.10) 1.00 Å^−1^ and 1.00 (0.20) 2.00 Å^−1^, together with *s* ∈ {2.50, 3.00, 3.50, 4.00, 5.00, 6.00} Å^−1^.

(vi) ITCi: *s* ∈ [0.00, 1.50 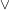 2.00] Å^−1^ in a grid Δ*s* 0.00 (0.01) 0.20 Å^−1^, 0.20 (0.02) 0.50 Å^−1^, 0.50 (0.05) 0.70 Å^−1^ and 0.70 (0.10) 1.50 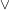 2.00 Å^−1^ + {0.25, 0.35, 0.45} Å^−1^.

(vii) RRGi: *s* ∈ [0.00, 6.00] Å^−1^ having the same grid as DTi.

(viii) WSBJi: *s* ∈ [0.00, 2.00] Å^−1^ in a grid Δ*s* 0.00 (0.01) 0.20 Å^−1^, 0.20 (0.02) 0.50 Å^−1^, 0.50 (0.05) 0.70 Å^−1^ and 0.70 (0.10) 2.00 Å^−1^ + {0.25, 0.35, 0.45} Å^−1^ and {2.50, 3.00, 3.50, 4.00, 5.00, 6.00} Å^−1^. In GT-I this was denoted as the IUCr grid.

(ix) MCi: *s* ∈ [0.00, 10.00] Å^−1^ in a grid Δ*s*: 0.00 (0.05) 10.00 Å^−1^.

(x) Yetali: *s* ∈ [0.00, 6.00] Å^−1^, Δ*s* having the IUCr grid.

(xi) OFFV1i: *s* ∈ [0.00, 6.00] Å^−1^, Δ*s* having the IUCr grid.

(xii) OFFV2i: *s* ∈ [0.00, 8.00] Å^−1^ in a grid Δ*s* 0.00 (0.01) 8.00 Å^−1^.

## 5. Results

The parameters of the final models for all data sets are presented in the supporting information.

**Figure 1:**
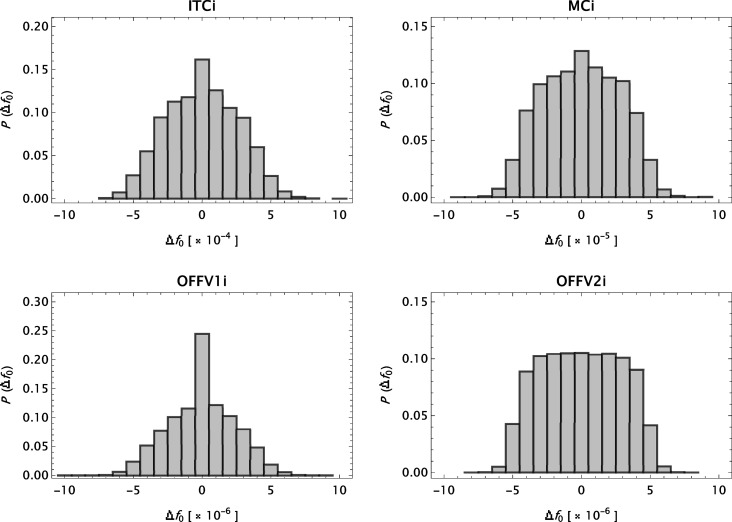
Histograms showing the distributions of deviations 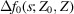 for various compilations. For ITCi, data point No. 97, representing Tl^3+^, has been omitted.

**Figure 2:**
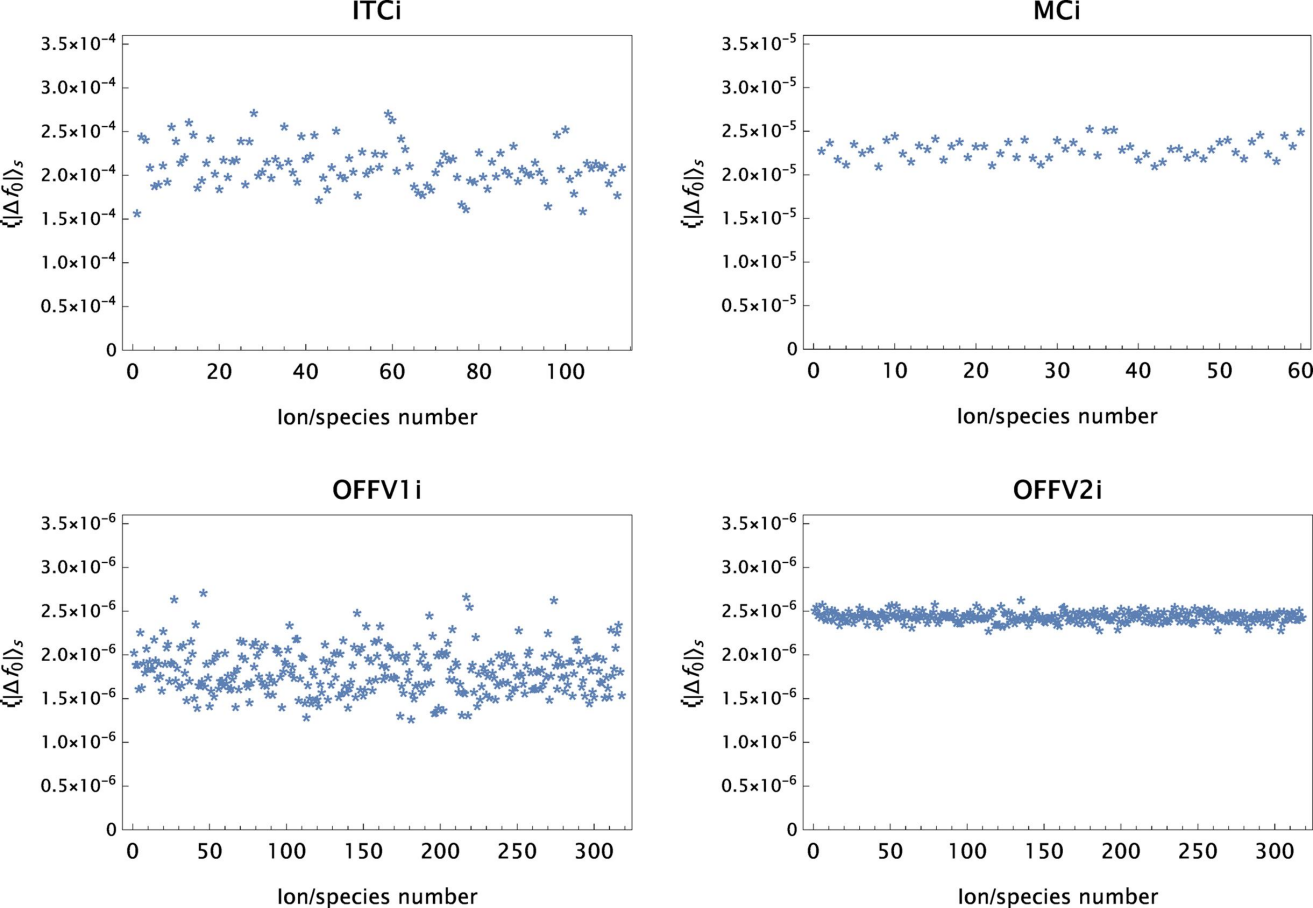
Examples of the variation of 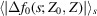 for the cases shown in Fig. 1 ▸.

The quality of the analytical modelling is evaluated in three different ways. (i) When the original data have a common precision, statistical measures are calculated (Table 4 ▸). In all cases the differences between the original data points and the model calculations are as expected. The rounding of form-factor values to the actual data precision may be regarded as a stochastic process described by a uniform statistical distribution. (ii) Form factors are calculated at the actual *s* grids based on the refined models and rounded to the same precision as the original data. The differences in the last significant digit are then compared. The results are presented in Table 5 ▸. We see that 96.1% of all modelled form factors exactly reproduce the underlying data. (iii) The distributions of errors {Δ*f*_0_ = *f*_0_(data) − *f*_0_(model)} [presented as histograms in Fig. 1 ▸ for four different data compilations, together with the corresponding graphical presentations of Δ*f*_0_(*s*) for the same cases as shown in Fig. 2 ▸] also verify that the accuracy of the modelling is determined by the precision (and inherent rounding) of the original data.

**Figure 3:**
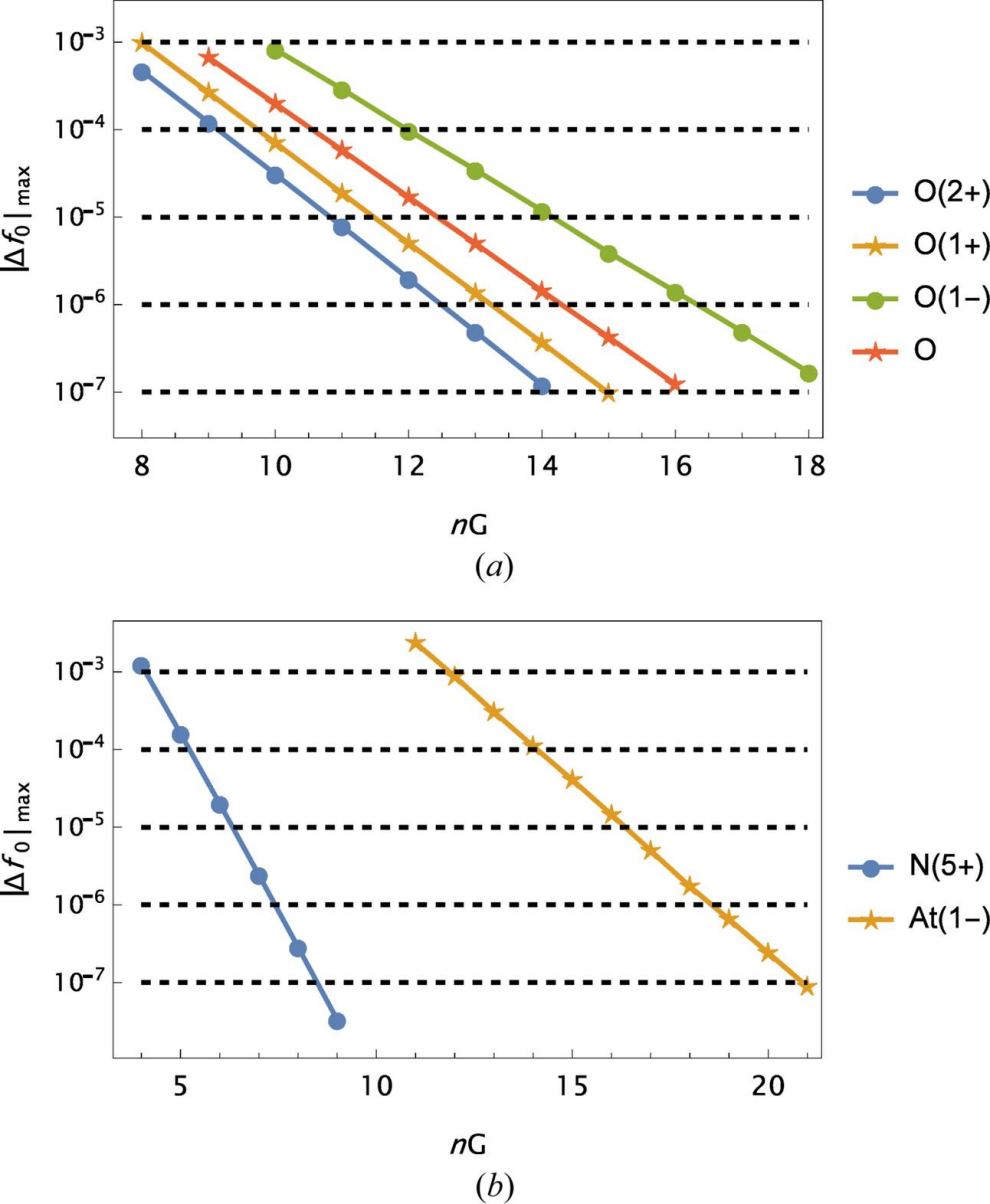
Examples of the variation of 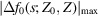 with the number of Gaussians in the analytical model, based on the original OFFV2i data. (*a*) Ions of oxygen, including the neutral atom. (*b*) Fastest and slowest development. For the neutral oxygen atom, data from Olukayode *et al.* (2023*a* ▸) are used.

An interesting feature is revealed in Fig. 3 ▸. Generally, for a given atomic number fewer Gaussians are needed in the modelling when Δ*Z* = *Z* − *Z*_0_ becomes more positive, *i.e.* for cations with an increasing net charge.

**Figure 4:**
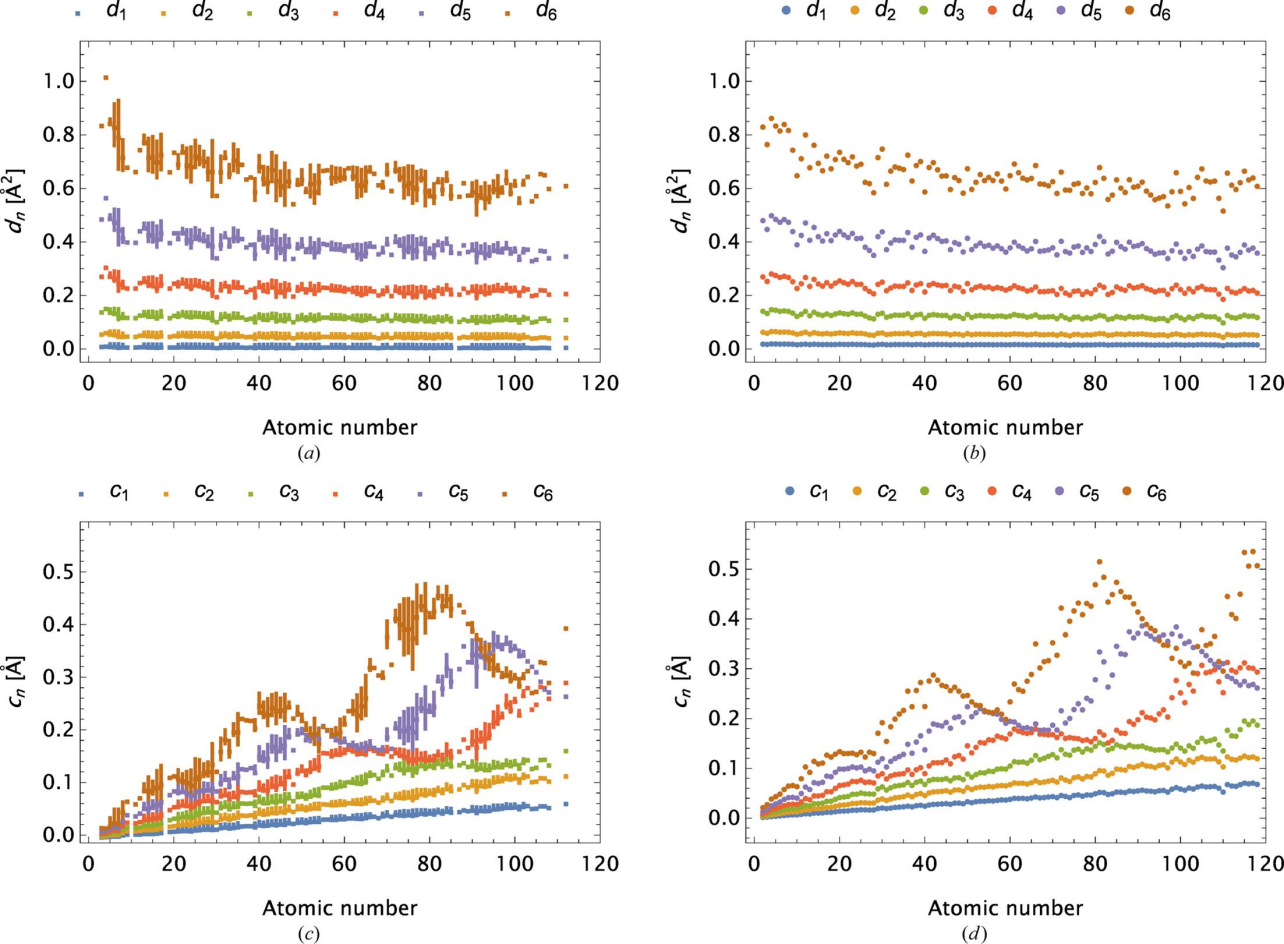
(*a*) and (*c*) Parameters *d*_*n*_ and *c*_*n*_ associated with OFFV2i; *n* = 1, …, 6. (*b*) and (*d*) Parameters *d*_*n*_ and *c*_*n*_ associated with neutral atoms included for comparison. In the last case, the parameters emerge from modelling of the extended data set provided by Volkov (*cf.* GT-I), rounded to a precision of 1 × 10^−5^.

In Fig. 4 ▸ the parameters *c*_*n*_ and *d*_*n*_ for *n* = 1, …, 6 are depicted for ions and neutral atoms based on OFFV2i and OFFV2 data, both rounded to a precision of 1 × 10^−5^. The ions are grouped according to their atomic number and, in the case of multiple occurrences, lines spanning the parameter values are used for plot markers. One readily observes the resemblance between this pair of figures. The parameters are organized according to increasing values of *d*, *i.e.* *d*_*n*_ < *d*_*n*+1_, and the values presented have the largest impact on the high-*s* value form factors, for which only small differences are expected between the neutral atoms and their associated ions.

Parameter values for oxygen and its ions from the OFFV2i analysis are explicitly given in Table 6 ▸. The main differences are linked to the Gaussians with the largest *d* values. Amplitudes typically increase and additional Gaussians, which appear in the models when Δ*Z* decreases, involve *large d* values and thus only influence form factors when evaluated for *small s* values.

## 6. Discussion

A few points are worth highlighting.

ITiiii: The compilation by Ibers (1962 ▸), which is documented in great detail, is built of contributions from several other authors. The presentation is, however, associated with a specific *s* grid, not always comprising the grids in the original reports. This is, among others, the case for the Watson & Freeman (1961 ▸) form-factor data (denoted SX-67 by Ibers). Slightly different parameter values are obtained, *e.g.* for the ions of nickel, based on the Ibers presentation compared with the one by Watson & Freeman (1961 ▸). In another case, ion S^2−^ (denoted AX-46) form-factor data are rounded from the original source (Tomiie & Stam, 1958 ▸) to fit the chosen *s* grid. An analysis of the original data set resulted in a slightly better fit than found in the ITiiii analysis. Generally, interpolated data sets give rise to larger residuals following the model refinements. The present fits to the inverse Mott–Bethe formula for C_val_, Zr^4+^ and Hg^2+^ are, for some reason, of poorer quality than the fits for the other ions.

ITCi: The form-factor data of Maslen *et al.* (1992 ▸) are, with the exception of those for H^−^, a copy of those first presented by Cromer & Waber (1974 ▸). This set is an original calculation not published elsewhere [*i.e.* not linked with the form factors of Cromer & Waber (1964 ▸)]. Identical parameter sets based on the traditional model of equation (3), S[4G + *c*], are provided in both these editions of *International Tables* (despite the change in the data for H^−^). It has further become evident that the published parameters for Ru^4+^ and Bi^5+^ are in error, leading to *e.g.* absolute deviations of, respectively, 3.0 and 16.2 for *s* = 2.0 Å^−1^. Excluding these ions from a statistical analysis based on the traditional model adopted in *International Tables* leads to 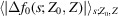 = 2.78 × 10^−3^ and 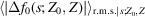 = 5.17 × 10^−3^, one order of magnitude larger than the values obtained in the present MB modelling. In this analysis the form factors of Tl^3+^ exhibit the most prominent deviations, Δ*f*_0_ ∈ [0.002–0.003], occurring for *s* ∈ [0.01, 0.06] Å^−1^. Waasmaier & Kirfel (1995 ▸) analysed the data of Maslen *et al.* (1992 ▸) in model S[5G + *c*]. They extended the data to *s*_max_ = 6.00 Å^−1^ by using data for neutral atoms for *s* > 2.00 Å^−1^ (or *s* > 1.50 Å^−1^), ‘*because scattering from valence electrons can be neglected*’ (Waasmaier & Kirfel, 1995 ▸). By applying their 11-parameter models for the restricted ranges actually published, one observes statistical measures a factor of two worse than found using nine-parameter models (Cromer & Waber, 1974 ▸). Altogether, it seems that a general update of the form-factor data for ions in *International Tables* is appropriate.

**Figure 5:**
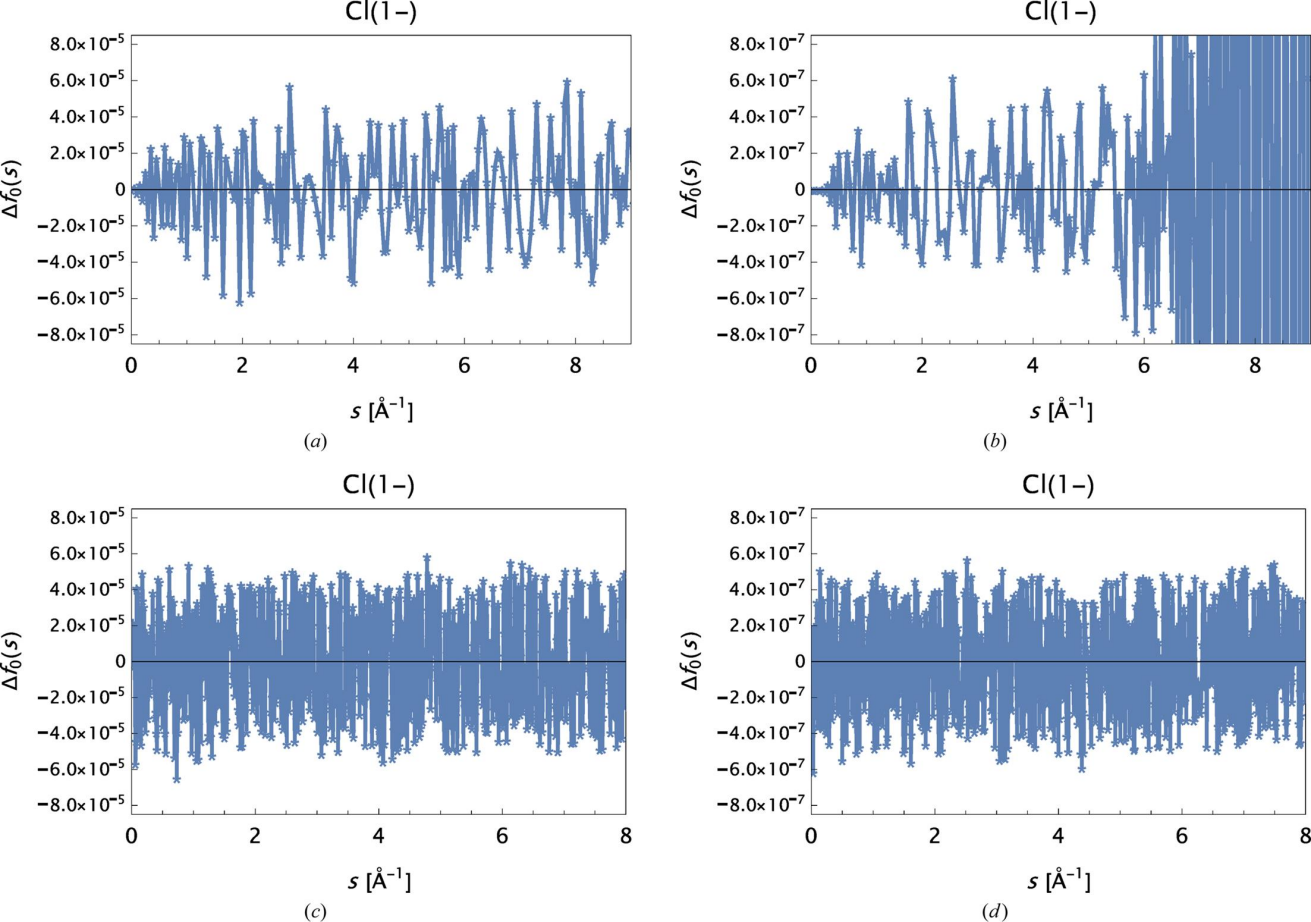
Δ*f*_0_(*s*) for Cl^−^. (*a*) and (*b*) Data from MCi, rounded to precisions (*a*) 10^−4^ and (*b*) 10^−6^. (*c*) and (*d*) An identical selection based on OFFV2i data.

MCi: The analysis reveals oscillations in Δ*f*_0_(*s*) for approximately *s* ≥ 5 Å^−1^. These are most prominent for the valence states C_val_ and Si_val_ and all anions. Oscillations are also observed for most of the cations (occurring for *s* ≥ 1–2 Å^−1^), but in these cases the amplitudes are smaller by at least one order of magnitude. The oscillations *disappear* when the original data are rounded to a precision of 1 × 10^−4^. This is depicted for Cl^−^ in Fig. 5 ▸ with the corresponding OFFV2i analysis as a reference.

**Figure 6:**
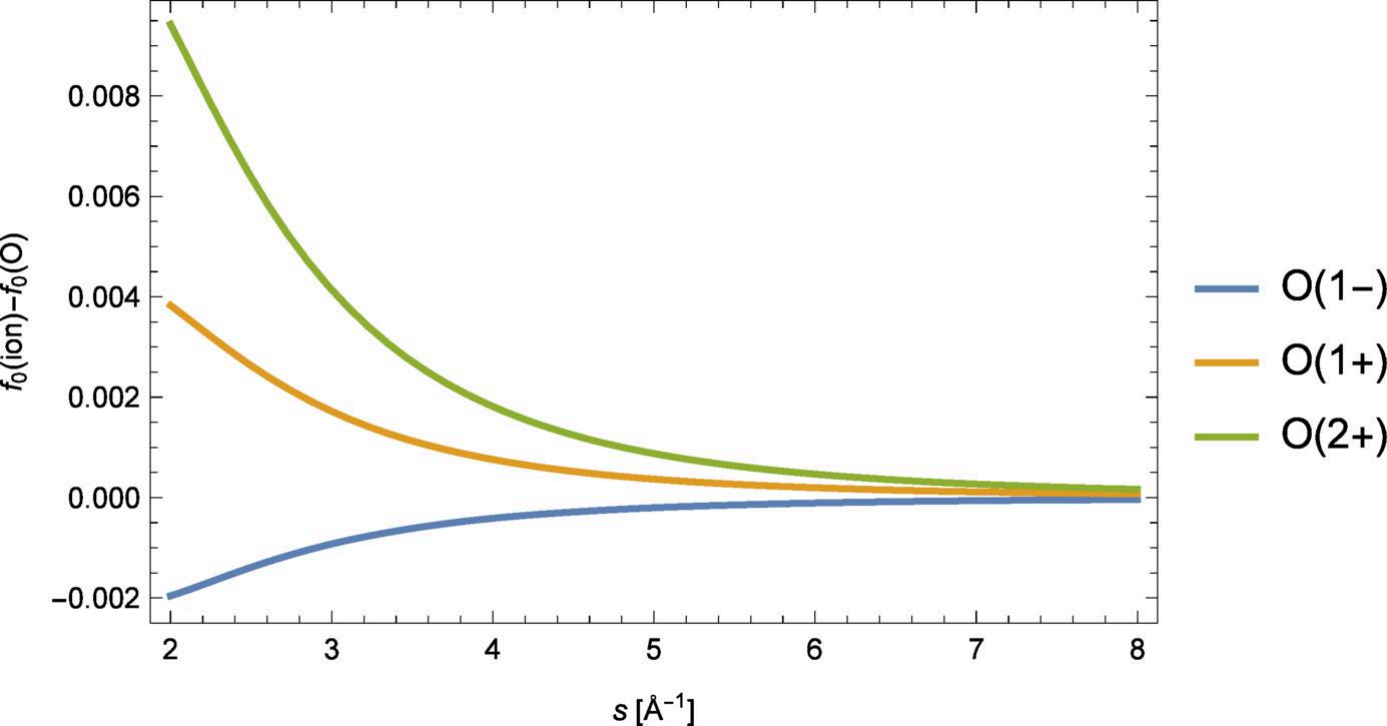
Deviation in form factors between selected ions of oxygen and neutral oxygen for *s* ≥ 2.0 Å^−1^. The figure is based on the inverse Mott–Bethe modelling of the extended data sets by Volkov (2023 ▸).

**Figure 7:**
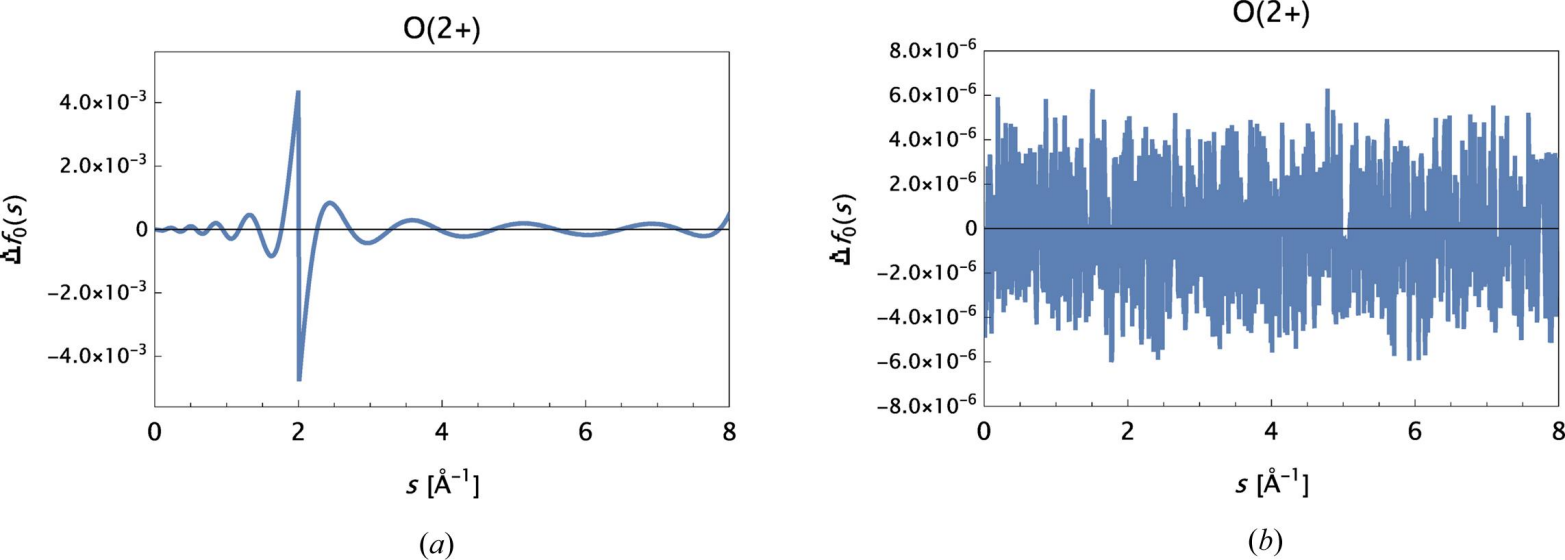
Δ*f*_0_(*s*) for O^2+^. (*a*) Constructed data based on OFFV2i for *s* ≤ 2.0 Å^−1^ and on OFFV2 for *s* > 2.0 Å^−1^. Best refined model: MB[8G + α]. (*b*) Data based on OFFV2i for the full range *s* ∈ [0.0, 8.0] Å^−1^. Final model: MB[13G + α].

General: Fig. 6 ▸ ▸ shows the differences in form-factor values of various ions of oxygen and oxygen itself, *e.g.* *f*_0_(*s*|O^2+^) − *f*_0_(*s*|O), for *s* ≥ 2.0 Å^−1^. The data are the sets provided by Volkov (2023 ▸) rounded to a precision of 1 × 10^−5^. The differences observed are roughly one to three orders of magnitude larger than the data precision. Thus, substitution of neutral-atom form-factor data when high-*s* value data are lacking for associated ions [as in Waasmaier & Kirfel (1995 ▸)] should be avoided. Fig. 7 ▸ shows the results of a detailed analysis for the ion O^2+^.

## 7. Concluding remarks

The modelling of form-factor data of neutral atoms accounted for in GT-I is also appropriate for ions. It gives improved analytical models compared with the traditional ones existing in the literature. The new models are easily implemented and can be applied in all cases where *e.g.* scattering factors are to be calculated. They are generally very accurate and flexible in such a way that original form-factor calculations, with different physical features incorporated (Schmidt & Weiss, 1979 ▸), are consistently reproduced. 

## Supplementary Material

List of data sets used. DOI: 10.1107/S2053273323010550/ae5139sup1.pdf

Data WFi. DOI: 10.1107/S2053273323010550/ae5139sup2.txt

Data ITiiii. DOI: 10.1107/S2053273323010550/ae5139sup3.txt

Data CLWi. DOI: 10.1107/S2053273323010550/ae5139sup4.txt

Data CMi. DOI: 10.1107/S2053273323010550/ae5139sup5.txt

Data DTi. DOI: 10.1107/S2053273323010550/ae5139sup6.txt

Data ITCi. DOI: 10.1107/S2053273323010550/ae5139sup7.txt

Data RRGi. DOI: 10.1107/S2053273323010550/ae5139sup8.txt

Data WSBJi. DOI: 10.1107/S2053273323010550/ae5139sup9.txt

Data MCi. DOI: 10.1107/S2053273323010550/ae5139sup10.txt

Data Yetali. DOI: 10.1107/S2053273323010550/ae5139sup11.txt

Data OFFV1i. DOI: 10.1107/S2053273323010550/ae5139sup12.txt

Data OFFV2i. DOI: 10.1107/S2053273323010550/ae5139sup13.txt

## Figures and Tables

**Table 1:** The number of species involved in the various data sets

Compilation	Neutral atoms	Valence states	Cations	Anions	Total
WFi	8		28		36
CLWi			50		50
CMi			73	5	78
DTi			19	3	22
ITCi		2	105	6	113
RRGi			42	5	47
WSBJi		2	4	2	8
MCi		2	53	5	60
OFFVi		2	310	6	318
ITiiii		1	75	10	86
Yetali			8	5	13

**Table 2:** Number of species with a parameter set involving *n*G Gaussians

Source	2G	3G	4G	5G	6G	7G	8G	9G	10G	11G	12G	13G	14G	15G	16G	17G	18G
WFi		1	14	13	8												
CLWi		1	3	4	22	20											
CMi		2	5	27	31	10	3										
DTi					2	4	6	6	4								
ITCi		2	5	16	47	32	10	1									
RRGi						3	5	27	8	4							
WSBJi					1	1	2			1	3						
MCi								2	1	5	18	21	9	3	1		
OFFV1i				1	3	4	10	38	100	101	44	11	6				
OFFV2i							2	3	2	7	23	61	105	86	24	3	2
ITiiii	7	12	33	28	3	1	2										
Yetali							3	4	3	3							

**Table 3:** Basic information related to the compilations The data of ITiiii and Yetali have variable precisions. For further comments regarding precision, see the text.

Compilation	Precision	Grid points	Form factors
WFi	1 × 10^−2^	21	756
CLWi	1 × 10^−2^	200	10 000
CMi	1 × 10^−3^	151	11 778
DTi	1 × 10^−3^	27	594
ITCi	1 × 10^−3^	51, 56	6223
RRGi	1 × 10^−4^	27	1269
WSBJi	1 × 10^−4^	62	496
MCi	1 × 10^−4^ †	201	12 060
OFFV1i	1 × 10^−5^	62	19 716
OFFV2i	1 × 10^−5^ ‡	801	254 718
ITiiii	1 × 10^−(1,2,3,4)^	12–24	1610
Yetali	1 × 10^−(2,3,4,5)^	62	806

† Original data have a precision of 1 × 10^−9^.

‡ Original data have a precision of 1 × 10^−10^.

**Table 4:** Statistical properties for compilations having a fixed precision

Compilation	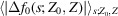	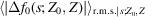	|Δ*f*_0_(*s*;*Z*_0_, *Z*)|_max_
WFi	1.59 × 10^−3^	2.06 × 10^−3^	7 × 10^−3^
CLWi	2.38 × 10^−3^	2.79 × 10^−3^	8 × 10^−3^
CMi	2.37 × 10^−4^	2.79 × 10^−4^	8 × 10^−4^
DTi	1.20 × 10^−4^	1.81 × 10^−4^	9 × 10^−4^
ITCi	2.14 × 10^−4^	2.71 × 10^−4^	3 × 10^−3^
RRGi	1.07 × 10^−5^	1.70 × 10^−5^	7 × 10^−5^
WSBJi	2.00 × 10^−5^	2.56 × 10^−5^	7 × 10^−5^
MCi	2.31 × 10^−5^	2.75 × 10^−5^	1 × 10^−4^
OFFV1i	1.83 × 10^−6^	2.37 × 10^−6^	1 × 10^−5^
OFFV2i	2.45 × 10^−6^	2.86 × 10^−6^	8 × 10^−6^

**Table 5:** Absolute deviations from the original form-factor values using the model calculations amount to 0 (no deviation), 1, or 2 and 3 in the last significant figure of the original data The incidences for all compilations are given as percentages. For ITiiii, species 10, 78 and 82 (C_val_, Zr^4+^ and Hg^2+^, respectively) are omitted from the calculation. See also Section 6.

Compilation	0	1	2 and 3
WFi	98.3	1.7	
CLWi	96.7	3.3	
CMi	96.0	4.0	
DTi	97.6	2.4	
ITCi	95.8	4.1	0.1
RRGi	98.3	1.7	
WSBJi	96.2	3.8	
MCi	95.9	4.1	
OFFV1i	97.0	3.0	
OFFV2i	96.1	3.9	
ITiiii	93.0	6.7	0.3
Yetali	92.4	7.3	0.3

**Table 6:** Parameters associated with oxygen and associated ions α and *c_i_* are in Å, *d_i_* in Å^2^. Actual data sets are OFFV2 and OFFV2i. Final models are MB[13G + α] for O^2+^ and O^1+^, MB[14G + α] for O and MB[16G + α] for O^1−^.

	O^2+^	O^1+^	O	O^1−^
α	0.00061 (0.00006)	0.00075 (0.00002)	0.000922 (0.000012)	0.00095 (0.00004)
				
*d*_1_	0.0159 (0.0015)	0.0167 (0.0005)	0.0179 (0.0002)	0.0164 (0.0007)
*d*_2_	0.055 (0.005)	0.0582 (0.0015)	0.0625 (0.0008)	0.057 (0.002)
*d*_3_	0.120 (0.010)	0.132 (0.003)	0.140 (0.002)	0.127 (0.005)
*d*_4_	0.231 (0.018)	0.252 (0.005)	0.267 (0.004)	0.239 (0.009)
*d*_5_	0.40 (0.03)	0.449 (0.008)	0.473 (0.008)	0.417 (0.015)
*d*_6_	0.67 (0.04)	0.781 (0.013)	0.816 (0.015)	0.70 (0.03)
*d*_7_	1.12 (0.05)	1.34 (0.02)	1.38 (0.03)	1.17 (0.04)
*d*_8_	1.89 (0.06)	2.28 (0.04)	2.30 (0.06)	1.93 (0.06)
*d*_9_	3.16 (0.09)	3.83 (0.06)	3.80 (0.10)	3.20 (0.10)
*d*_10_	5.24 (0.15)	6.40 (0.10)	6.21 (0.15)	5.24 (0.14)
*d*_11_	8.6 (0.3)	10.79 (0.16)	10.3 (0.2)	8.7 (0.2)
*d*_12_	13.9 (0.5)	18.3 (0.3)	17.5 (0.4)	14.7 (0.4)
*d*_13_	23.2 (0.8)	32.2 (0.5)	30.3 (0.6)	25.5 (0.6)
*d*_14_			54.8 (0.9)	45.5 (1.1)
*d*_15_				84 (2)
*d*_16_				164 (4)
				
*c*_1_	0.0039 (0.0003)	0.00477 (0.00013)	0.00585 (0.00008)	0.0060 (0.0003)
*c*_2_	0.0074 (0.0006)	0.0092 (0.0002)	0.01126 (0.00015)	0.0115 (0.0005)
*c*_3_	0.0117 (0.0008)	0.0149 (0.0003)	0.0180 (0.0003)	0.0184 (0.0007)
*c*_4_	0.0170 (0.0010)	0.0225 (0.0003)	0.0272 (0.0004)	0.0275 (0.0009)
*c*_5_	0.0234 (0.0011)	0.0336 (0.0005)	0.0411 (0.0007)	0.0409 (0.0013)
*c*_6_	0.0344 (0.0011)	0.0525 (0.0009)	0.0642 (0.0016)	0.062 (0.002)
*c*_7_	0.0569 (0.0017)	0.0883 (0.0017)	0.105 (0.003)	0.100 (0.004)
*c*_8_	0.101 (0.003)	0.153 (0.003)	0.174 (0.006)	0.167 (0.006)
*c*_9_	0.167 (0.004)	0.241 (0.003)	0.272 (0.007)	0.275 (0.007)
*c*_10_	0.214 (0.004)	0.306 (0.003)	0.375 (0.005)	0.412 (0.008)
*c*_11_	0.177 (0.005)	0.261 (0.004)	0.411 (0.006)	0.533 (0.007)
*c*_12_	0.072 (0.007)	0.117 (0.004)	0.315 (0.008)	0.581 (0.008)
*c*_13_	0.0085 (0.0018)	0.0161 (0.0013)	0.142 (0.007)	0.519 (0.009)
*c*_14_			0.0228 (0.0019)	0.362 (0.009)
*c*_15_				0.165 (0.008)
*c*_16_				0.030 (0.003)
