# Supplementary material for: Analytical models representing X-ray form factors of ions
Source: Acta Crystallogr A Found Adv. 2024 Jan 1;80(Pt 1):129–36. doi: 10.1107/S2053273323010550 (PMC10833360; doi:10.1107/S2053273323010550)
Supplement: Supplementary file 1 [file a-80-00129-sup1.pdf]

Acta Cryst A FOUNDATIONS ADVANCES

Volume 80 (2024)

Supporting information for article:

Analytical models representing X-ray form factors of ions

Gunnar Thorkildsen

Ions / species examined

Watson & Freeman (1961)

Code: **WFi**

International Tables Vol. III (1962)

Code: **ITiiii**

Cromer, Larson & Waber (1963)

Code: **CLWi**

Cromer & Mann (1968)

Code: **CMi**

Doyle & Turner (1968)

Code: **DTi**

International Tables Vol. IV (1974) & Vol. C (1992)

Code: **ITCi**

Rez, Rez & Grant (1994)

Code: **RRGi**

Wang , Smith, Bunge & Jáuregui (1996)

Code: **WSBJi**

Macchi & Coppens (2001)

Code: **MCi**

Yonekura et al (2018)

Code: **Yetali**

Olukayode, Froese Fischer & Volkov (2023)

Code: **OFFVi**

WFi

Number	Species	Number	Species
1	Sc	19	Mn^{2+}
2	Sc^{1+}	20	Mn^{3+}
3	Sc^{2+}	21	Fe
4	Sc^{3+}	22	Fe^{1+}
5	Ti	23	Fe^{2+}
6	Ti^{1+}	24	Fe^{3+}
7	Ti^{2+}	25	Fe^{4+}
8	Ti^{3+}	26	Co
9	V	27	Co^{1+}
10	V^{1+}	28	Co^{2+}
11	V^{2+}	29	Co^{3+}
12	V^{3+}	30	Ni
13	Cr	31	Ni^{1+}
14	Cr^{1+}	32	Ni^{2+}
15	Cr^{2+}	33	Ni^{3+}
16	Cr^{3+}	34	Cu^{1+}
17	Mn	35	Cu^{2+}
18	Mn^{1+}	36	Cu^{3+}

ITiiii

Number	Species	Number	Species	Number	Species
1	H^{1-}	31	Cl^{1-}	61	Cu^{1+}
2	He^{1-}	32	K^{1+}	62	Cu^{2+}
3	Li^{1+}	33	Ca^{1+}	63	Cu^{3+}
4	Li^{1-}	34	Ca^{2+}	64	Zn^{2+}
5	Be^{1+}	35	Sc^{1+}	65	Ga^{1+}
6	Be^{2+}	36	Sc^{2+}	66	Ga^{3+}
7	B^{1+}	37	Sc^{3+}	67	Ge^{2+}
8	B^{2+}	38	Ti^{1+}	68	Ge^{4+}
9	B^{3+}	39	Ti^{2+}	69	As^{1+}
10	C_{val}	40	Ti^{3+}	70	As^{2+}
11	C^{2+}	41	V^{1+}	71	As^{3+}
12	C^{3+}	42	V^{2+}	72	As^{5+}
13	C^{4+}	43	V^{3+}	73	Se^{6+}
14	N^{3+}	44	Cr^{1+}	74	Br^{7+}
15	N^{4+}	45	Cr^{2+}	75	Br^{1-}
16	N^{1-}	46	Cr^{3+}	76	Kr^{8+}
17	O^{1+}	47	Mn^{1+}	77	Rb^{1+}
18	O^{2+}	48	Mn^{2+}	78	Zr^{4+}
19	O^{3+}	49	Mn^{3+}	79	Mo^{1+}
20	O^{1-}	50	Mn^{4+}	80	Ag^{1+}
21	F^{1-}	51	Fe^{1+}	81	Au^{1+}
22	Na^{1+}	52	Fe^{2+}	82	Hg^{2+}
23	Mg^{2+}	53	Fe^{3+}	83	Tl^{1+}
24	Al^{1+}	54	Fe^{4+}	84	Tl^{3+}
25	Al^{2+}	55	Co^{1+}	85	Pb^{3+}
26	Al^{3+}	56	Co^{2+}	86	U^{6+}
27	Si^{3+}	57	Co^{3+}		
28	Si^{4+}	58	Ni^{1+}		
29	S^{1-}	59	Ni^{2+}		
30	S^{2-}	60	Ni^{3+}		

CLWi

Number	Species	Number	Species
1	Li^{1+}	26	Pm^{3+}
2	Na^{1+}	27	Sm^{3+}
3	Mg^{2+}	28	Eu^{2+}
4	Al^{3+}	29	Eu^{3+}
5	Si^{4+}	30	Gd^{3+}
6	K^{1+}	31	Tb^{3+}
7	Ca^{2+}	32	Tb^{4+}
8	Sc^{3+}	33	Dy^{3+}
9	Ni^{2+}	34	Ho^{3+}
10	Cu^{1+}	35	Er^{3+}
11	Cu^{2+}	36	Tm^{3+}
12	Zn^{2+}	37	Yb^{2+}
13	Rb^{1+}	38	Yb^{3+}
14	Sr^{2+}	39	Lu^{3+}
15	Y^{3+}	40	Au^{1+}
16	Zr^{4+}	41	Hg^{2+}
17	Pd^{2+}	42	Tl^{1+}
18	Ag^{1+}	43	Bi^{3+}
19	Cd^{2+}	44	Fr^{1+}
20	La^{3+}	45	Ra^{2+}
21	Ce^{3+}	46	Ac^{3+}
22	Ce^{4+}	47	Th^{4+}
23	Pr^{3+}	48	U^{4+}
24	Pr^{4+}	49	Np^{4+}
25	Nd^{3+}	50	Pu^{4+}

CMi

Number	Species	Number	Species	Number	Species
1	Li^{1+}	31	Zn^{2+}	61	Ce^{3+}
2	Be^{2+}	32	Ga^{3+}	62	Ce^{4+}
3	O^{1-}	33	Br^{1-}	63	Pr^{3+}
4	F^{1-}	34	Rb^{1+}	64	Pr^{4+}
5	Na^{1+}	35	Sr^{2+}	65	Nd^{3+}
6	Mg^{2+}	36	Y^{3+}	66	Pm^{3+}
7	Al^{3+}	37	Zr^{4+}	67	Sm^{3+}
8	Si^{4+}	38	Nb^{3+}	68	Eu^{2+}
9	Cl^{1-}	39	Nb^{5+}	69	Eu^{3+}
10	K^{1+}	40	Mo^{3+}	70	Gd^{3+}
11	Ca^{2+}	41	Mo^{5+}	71	Tb^{3+}
12	Sc^{3+}	42	Mo^{6+}	72	Dy^{3+}
13	Ti^{3+}	43	Ru^{3+}	73	Ho^{3+}
14	Ti^{4+}	44	Ru^{4+}	74	Er^{3+}
15	V^{2+}	45	Rh^{3+}	75	Tm^{3+}
16	V^{3+}	46	Rh^{4+}	76	Yb^{2+}
17	V^{5+}	47	Pd^{2+}	77	Yb^{3+}
18	Cr^{2+}	48	Pd^{4+}	78	Lu^{3+}
19	Cr^{3+}	49	Ag^{1+}		
20	Mn^{2+}	50	Ag^{2+}		
21	Mn^{3+}	51	Cd^{2+}		
22	Mn^{4+}	52	In^{3+}		
23	Fe^{2+}	53	Sn^{2+}		
24	Fe^{3+}	54	Sn^{4+}		
25	Co^{2+}	55	Sb^{3+}		
26	Co^{3+}	56	Sb^{5+}		
27	Ni^{2+}	57	I^{1-}		
28	Ni^{3+}	58	Cs^{1+}		
29	Cu^{1+}	59	Ba^{2+}		
30	Cu^{2+}	60	La^{3+}		

DTi			
Number	Species	Number	Species
1	Li^{1+}	12	Co^{2+}
2	Be^{2+}	13	Ni^{2+}
3	Na^{1+}	14	Cu^{1+}
4	Mg^{2+}	15	Zn^{2+}
5	Cl^{1-}	16	Br^{1-}
6	K^{1+}	17	Rb^{1+}
7	Ca^{2+}	18	Sr^{2+}
8	V^{2+}	19	Sn^{2+}
9	Mn^{2+}	20	Sn^{4+}
10	Fe^{2+}	21	I^{1-}
11	Fe^{3+}	22	Cs^{1+}

ITCi

Number	Species	Number	Species	Number	Species	Number	Species
1	H^{1-}	31	Ni^{2+}	61	Sb^{5+}	91	Pt^{4+}
2	Li^{1+}	32	Ni^{3+}	62	I^{1-}	92	Au^{1+}
3	Be^{2+}	33	Cu^{1+}	63	Cs^{1+}	93	Au^{3+}
4	C_{val}	34	Cu^{2+}	64	Ba^{2+}	94	Hg^{1+}
5	O^{1-}	35	Zn^{2+}	65	La^{3+}	95	Hg^{2+}
6	F^{1-}	36	Ga^{3+}	66	Ce^{3+}	96	Tl^{1+}
7	Na^{1+}	37	Ge^{4+}	67	Ce^{4+}	97	Tl^{3+}
8	Mg^{2+}	38	Br^{1-}	68	Pr^{3+}	98	Pb^{2+}
9	Al^{3+}	39	Rb^{1+}	69	Pr^{4+}	99	Pb^{4+}
10	Si_{val}	40	Sr^{2+}	70	Nd^{3+}	100	Bi^{3+}
11	Si^{4+}	41	Y^{3+}	71	Pm^{3+}	101	Bi^{5+}
12	Cl^{1-}	42	Zr^{4+}	72	Sm^{3+}	102	Ra^{2+}
13	K^{1+}	43	Nb^{3+}	73	Eu^{2+}	103	Ac^{3+}
14	Ca^{2+}	44	Nb^{5+}	74	Eu^{3+}	104	Th^{4+}
15	Sc^{3+}	45	Mo^{3+}	75	Gd^{3+}	105	U^{3+}
16	Ti^{2+}	46	Mo^{5+}	76	Tb^{3+}	106	U^{4+}
17	Ti^{3+}	47	Mo^{6+}	77	Dy^{3+}	107	U^{6+}
18	Ti^{4+}	48	Ru^{3+}	78	Ho^{3+}	108	Np^{3+}
19	V^{2+}	49	Ru^{4+}	79	Er^{3+}	109	Np^{4+}
20	V^{3+}	50	Rh^{3+}	80	Tm^{3+}	110	Np^{6+}
21	V^{5+}	51	Rh^{4+}	81	Yb^{2+}	111	Pu^{3+}
22	Cr^{2+}	52	Pd^{2+}	82	Yb^{3+}	112	Pu^{4+}
23	Cr^{3+}	53	Pd^{4+}	83	Lu^{3+}	113	Pu^{6+}
24	Mn^{2+}	54	Ag^{1+}	84	Hf^{4+}		
25	Mn^{3+}	55	Ag^{2+}	85	Ta^{5+}		
26	Mn^{4+}	56	Cd^{2+}	86	W^{6+}		
27	Fe^{2+}	57	In^{3+}	87	Os^{4+}		
28	Fe^{3+}	58	Sn^{2+}	88	Ir^{3+}		
29	Co^{2+}	59	Sn^{4+}	89	Ir^{4+}		
30	Co^{3+}	60	Sb^{3+}	90	Pt^{2+}		

RRGi

Number	Species	Number	Species
1	Li^{1+}	26	Nb^{5+}
2	Be^{2+}	27	Mo^{6+}
3	O^{2-}	28	Pd^{2+}
4	F^{1-}	29	Ag^{2+}
5	Na^{1+}	30	Cd^{2+}
6	Mg^{2+}	31	I^{1-}
7	Al^{3+}	32	Cs^{1+}
8	Cl^{1-}	33	Ba^{2+}
9	K^{1+}	34	La^{3+}
10	Ca^{2+}	35	Ce^{4+}
11	Sc^{3+}	36	Pr^{3+}
12	Ti^{4+}	37	Nd^{3+}
13	V^{5+}	38	Sm^{3+}
14	Cr^{4+}	39	Eu^{3+}
15	Mn^{2+}	40	Gd^{3+}
16	Fe^{2+}	41	Tb^{3+}
17	Co^{2+}	42	Dy^{3+}
18	Ni^{2+}	43	Ho^{3+}
19	Cu^{2+}	44	Er^{3+}
20	Zn^{2+}	45	Tm^{3+}
21	Br^{1-}	46	Yb^{3+}
22	Rb^{1+}	47	Lu^{3+}
23	Sr^{2+}		
24	Y^{3+}		
25	Zr^{4+}		

**WSBJi**	
Number	Species
1	Li^{1+}
2	Be^{2+}
3	C_{val}
4	F^{1-}
5	Na^{1+}
6	Mg^{2+}
7	Si_{val}
8	Cl^{1-}

MCi

Number	Species	Number	Species
1	Li^{1+}	31	Ni^{3+}
2	Be^{2+}	32	Cu^{1+}
3	C_{val}	33	Cu^{2+}
4	O^{1-}	34	Zn^{2+}
5	F^{1-}	35	Ga^{3+}
6	Na^{1+}	36	Ge^{4+}
7	Mg^{2+}	37	Br^{1-}
8	Al^{3+}	38	Rb^{1+}
9	Si_{val}	39	Sr^{2+}
10	Si^{4+}	40	Y^{3+}
11	Cl^{1-}	41	Zr^{4+}
12	K^{1+}	42	Nb^{3+}
13	Ca^{2+}	43	Nb^{5+}
14	Sc^{3+}	44	Mo^{3+}
15	Ti^{2+}	45	Mo^{6+}
16	Ti^{3+}	46	Ru^{3+}
17	Ti^{4+}	47	Ru^{4+}
18	V^{2+}	48	Rh^{3+}
19	V^{3+}	49	Rh^{4+}
20	V^{5+}	50	Pd^{2+}
21	Cr^{2+}	51	Pd^{4+}
22	Cr^{3+}	52	Ag^{1+}
23	Mn^{2+}	53	Ag^{2+}
24	Mn^{3+}	54	Cd^{2+}
25	Mn^{4+}	55	In^{3+}
26	Fe^{2+}	56	Sn^{2+}
27	Fe^{3+}	57	Sn^{4+}
28	Co^{2+}	58	Sb^{3+}
29	Co^{3+}	59	Sb^{5+}
30	Ni^{2+}	60	I^{1-}

**Yetali**	
Number	Species
1	C^{1+}
2	C^{1-}
3	N^{1+}
4	N^{1-}
5	O^{1+}
6	O^{1-}
7	P^{1+}
8	P^{1-}
9	P^{2+}
10	S^{1+}
11	S^{1-}
12	S^{2+}
13	S^{3+}

OFFVi

Number	Species	Number	Species	Number	Species	Number	Species	Number	Species	Number	Species	Number	Species	Number	Species
1	Li^{1+}	41	Cl^{2+}	81	Co^{2+}	121	Zr^{4+}	161	In^{3+}	201	Tb^{4+}	241	Ir^{3+}	281	U^{6+}
2	Be^{2+}	42	Cl^{3+}	82	Co^{3+}	122	Nb^{2+}	162	Sn^{2+}	202	Dy^{2+}	242	Ir^{4+}	282	Np^{3+}
3	B^{1+}	43	Cl^{4+}	83	Co^{4+}	123	Nb^{3+}	163	Sn^{4+}	203	Dy^{3+}	243	Ir^{5+}	283	Np^{4+}
4	B^{2+}	44	Cl^{5+}	84	Co^{5+}	124	Nb^{4+}	164	Sb^{3+}	204	Ho^{3+}	244	Ir^{6+}	284	Np^{5+}
5	B^{3+}	45	Cl^{6+}	85	Ni^{1+}	125	Nb^{5+}	165	Sb^{5+}	205	Er^{3+}	245	Pt^{2+}	285	Np^{6+}
6	C^{1+}	46	Cl^{7+}	86	Ni^{2+}	126	Mo^{1+}	166	Te^{2+}	206	Tm^{2+}	246	Pt^{4+}	286	Np^{7+}
7	C^{2+}	47	Cl^{1-}	87	Ni^{3+}	127	Mo^{2+}	167	Te^{4+}	207	Tm^{3+}	247	Pt^{5+}	287	Pu^{3+}
8	C^{3+}	48	K^{1+}	88	Ni^{4+}	128	Mo^{3+}	168	Te^{5+}	208	Yb^{2+}	248	Pt^{6+}	288	Pu^{4+}
9	C^{4+}	49	Ca^{2+}	89	Cu^{1+}	129	Mo^{4+}	169	Te^{6+}	209	Yb^{3+}	249	Au^{1+}	289	Pu^{5+}
10	C_{val}	50	Sc^{1+}	90	Cu^{2+}	130	Mo^{5+}	170	I^{1+}	210	Lu^{3+}	250	Au^{2+}	290	Pu^{6+}
11	N^{1+}	51	Sc^{2+}	91	Cu^{3+}	131	Mo^{6+}	171	I^{3+}	211	Hf^{2+}	251	Au^{3+}	291	Pu^{7+}
12	N^{2+}	52	Sc^{3+}	92	Cu^{4+}	132	Tc^{1+}	172	I^{5+}	212	Hf^{3+}	252	Au^{5+}	292	Am^{2+}
13	N^{3+}	53	Ti^{2+}	93	Zn^{2+}	133	Tc^{2+}	173	I^{7+}	213	Hf^{4+}	253	Hg^{1+}	293	Am^{3+}
14	N^{4+}	54	Ti^{3+}	94	Ga^{1+}	134	Tc^{3+}	174	I^{1-}	214	Ta^{2+}	254	Hg^{2+}	294	Am^{4+}
15	N^{5+}	55	Ti^{4+}	95	Ga^{2+}	135	Tc^{4+}	175	Xe^{2+}	215	Ta^{3+}	255	Tl^{1+}	295	Am^{5+}
16	O^{1+}	56	V^{1+}	96	Ga^{3+}	136	Tc^{5+}	176	Xe^{4+}	216	Ta^{4+}	256	Tl^{3+}	296	Am^{6+}
17	O^{2+}	57	V^{2+}	97	Ge^{1+}	137	Tc^{6+}	177	Xe^{6+}	217	Ta^{5+}	257	Pb^{2+}	297	Cm^{3+}
18	O^{1-}	58	V^{3+}	98	Ge^{2+}	138	Tc^{7+}	178	Xe^{8+}	218	W^{1+}	258	Pb^{4+}	298	Cm^{4+}
19	F^{1-}	59	V^{4+}	99	Ge^{3+}	139	Ru^{1+}	179	Cs^{1+}	219	W^{2+}	259	Bi^{3+}	299	Bk^{3+}
20	Na^{1+}	60	V^{5+}	100	Ge^{4+}	140	Ru^{2+}	180	Ba^{2+}	220	W^{3+}	260	Bi^{5+}	300	Bk^{4+}
21	Mg^{2+}	61	Cr^{1+}	101	As^{2+}	141	Ru^{3+}	181	La^{2+}	221	W^{4+}	261	Po^{2+}	301	Cf^{2+}
22	Al^{1+}	62	Cr^{2+}	102	As^{3+}	142	Ru^{4+}	182	La^{3+}	222	W^{5+}	262	Po^{4+}	302	Cf^{3+}
23	Al^{3+}	63	Cr^{3+}	103	As^{5+}	143	Ru^{5+}	183	Ce^{2+}	223	W^{6+}	263	Po^{6+}	303	Cf^{4+}
24	Si^{1+}	64	Cr^{4+}	104	Se^{2+}	144	Ru^{6+}	184	Ce^{3+}	224	Re^{1+}	264	At^{1+}	304	Es^{2+}
25	Si^{2+}	65	Cr^{5+}	105	Se^{4+}	145	Ru^{7+}	185	Ce^{4+}	225	Re^{2+}	265	At^{3+}	305	Es^{3+}
26	Si^{3+}	66	Cr^{6+}	106	Se^{6+}	146	Ru^{8+}	186	Pr^{2+}	226	Re^{3+}	266	At^{5+}	306	Fm^{2+}
27	Si^{4+}	67	Mn^{1+}	107	Br^{1+}	147	Rh^{1+}	187	Pr^{3+}	227	Re^{4+}	267	At^{7+}	307	Fm^{3+}
28	Si_{val}	68	Mn^{2+}	108	Br^{3+}	148	Rh^{2+}	188	Pr^{4+}	228	Re^{5+}	268	At^{1-}	308	Md^{2+}
29	P^{1+}	69	Mn^{3+}	109	Br^{4+}	149	Rh^{3+}	189	Nd^{2+}	229	Re^{6+}	269	Fr^{1+}	309	Md^{3+}
30	P^{2+}	70	Mn^{4+}	110	Br^{5+}	150	Rh^{4+}	190	Nd^{3+}	230	Re^{7+}	270	Ra^{2+}	310	No^{2+}
31	P^{3+}	71	Mn^{5+}	111	Br^{7+}	151	Rh^{5+}	191	Pm^{3+}	231	Os^{1+}	271	Ac^{3+}	311	No^{3+}
32	P^{4+}	72	Mn^{6+}	112	Br^{1-}	152	Rh^{6+}	192	Sm^{2+}	232	Os^{2+}	272	Th^{2+}	312	Lr^{3+}
33	P^{5+}	73	Mn^{7+}	113	Kr^{2+}	153	Pd^{2+}	193	Sm^{3+}	233	Os^{3+}	273	Th^{3+}	313	Rf^{4+}
34	S^{1+}	74	Fe^{1+}	114	Rb^{1+}	154	Pd^{4+}	194	Eu^{2+}	234	Os^{4+}	274	Th^{4+}	314	Db^{5+}
35	S^{2+}	75	Fe^{2+}	115	Sr^{2+}	155	Ag^{1+}	195	Eu^{3+}	235	Os^{5+}	275	Pa^{3+}	315	Sg^{6+}
36	S^{3+}	76	Fe^{3+}	116	Y^{2+}	156	Ag^{2+}	196	Gd^{1+}	236	Os^{6+}	276	Pa^{4+}	316	Bh^{7+}
37	S^{4+}	77	Fe^{4+}	117	Y^{3+}	157	Ag^{3+}	197	Gd^{2+}	237	Os^{7+}	277	Pa^{5+}	317	Hs^{8+}
38	S^{5+}	78	Fe^{5+}	118	Zr^{1+}	158	Cd^{2+}	198	Gd^{3+}	238	Os^{8+}	278	U^{3+}	318	Cn^{2+}
39	S^{6+}	79	Fe^{6+}	119	Zr^{2+}	159	In^{1+}	199	Tb^{1+}	239	Ir^{1+}	279	U^{4+}		
40	Cl^{1+}	80	Co^{1+}	120	Zr^{3+}	160	In^{2+}	200	Tb^{3+}	240	Ir^{2+}	280	U^{5+}		
